# A starch- and ROS-regulating heat shock protein helps maintain male fertility in heat-stressed rice plants

**DOI:** 10.1093/plphys/kiad217

**Published:** 2023-04-13

**Authors:** Jiawen Chen

**Affiliations:** Plant Physiology, American Society of Plant Biologists, USA; John Innes Centre, Norwich Research Park, Norwich, NR4 7UH, UK

To maintain crop yields in our warming global climate, there is an urgent need to breed crops that are resilient to high temperatures. Heat stress severely affects rice (*Oryza sativa*), especially its reproductive stages, and can lead to significant crop losses. Among its physiological consequences, heat stress can increase reactive oxygen species (ROS) levels (Chen et al. 2022) and decrease starch accumulation in male reproductive tissues of various plants (De Storme and Geelen 2014). In mature rice pollen, starch is needed to support pollen germination and pollen tube growth, and starchless mutants are male sterile (Lee et al. 2016). Conceivably, reduced starch levels in pollen under heat stress could lead directly to male sterility, or perhaps reduced levels of starch in response to heat stress is an indirect consequence of general pollen dysfunction at high temperatures.

Starch biosynthesis involves the orchestration of many enzymes and non-enzymatic proteins, and the mechanism of starch granule initiation is poorly understood. In nonphotosynthetic tissues such as pollen, starch is synthesized in plastids called amyloplasts. *Os*FLO6 (FLOURY ENDOSPERM 6) is a non-enzymatic protein with a carbohydrate binding domain that is important for proper starch biosynthesis in rice leaves, endosperm, and pollen (Peng et al. 2014; Zhang et al. 2022). The loss of *Os*FLO6 in pollen reduces male fertility. In Arabidopsis (*Arabidopsis thaliana*), an ortholog of FLO6 is needed for starch granule initiation in the leaves, possibly by its direction of glucan substrates to a glycosyltransferase enzyme (Seung et al. 2017).

Heat stress responses in plants involve myriad physiological and molecular processes, such as the induction of heat shock proteins (HSPs) that aid in protein folding and stability by acting as chaperonins. HSP60s are one of the 5 major families of HSPs, with one of the best studied HSP60s being GroEL from *Escherichia coli*, which functions as a large homooligomeric complex. In plants, the chloroplast HSP60s, also known as chaperonin60s (Cpn60s), have α and β subunits, likely forming heterooligomeric complexes. However, different species have different numbers of Cpn60α and Cpn60β isoforms, and the exact oligomeric assembly of these subunits is not known. In rice, 3 *Cpn60α* and 3 *Cpn60β* genes have been identified, of which *OsCpn60α1*, *OsCpn60α2*/*TCD9*, and *OsCpn60β1* have been characterized (Kim et al. 2013; Jiang et al. 2014; Wu et al. 2020), all of which are crucial for chloroplast development.

In this issue of *Plant Physiology*, Lin et al. (2023) describe the previously uncharacterized gene *OsHSP60-3B*, which encodes a plastidial HSP and is important for pollen viability under heat stress. Using a radiation screen, the authors identified *oshsp60-3b* as a heat-sensitive, male sterile mutant, and its allelic identity was confirmed using map-based cloning and sequencing. As revealed by quantitative reverse transcription (qRT)-PCR and GUS staining, *OsHSP60-3B* is widely expressed across plant tissues. *OsHSP60-3B* expression increased over anther development and was induced by heat stress. However, in contrast to previously characterized *OsHSP60s*, the loss of *OsHSP60-3B* neither impacts the development of vegetative tissues nor influences the plant phenotype under ambient temperatures (22 to 28 °C). Instead, *OsHSP60-3B* had specific roles in regulating pollen starch synthesis and ROS under heat stress.

The *oshsp60-3b* mutant resembled the wild type under ambient temperatures but had pale anthers, inviable and shrunken pollen, and impaired pollen starch synthesis at high temperatures. This was seen under field conditions as well as in controlled environments. The early development of anther, tapetum, and microspore was not affected, suggesting heat stress specifically affected starch accumulation in the pollen at later stages. At pollen maturity, *oshsp60-3b* had smaller starch granules than the wild type at 28 °C and was missing starch entirely at 34 °C. Using a yeast two-hybrid screen for *Os*HSP60-3B interactors in the anther, the authors identified *Os*FLO6 as the only plastid-localized interaction candidate. They confirmed this interaction with yeast two-hybrid, split luciferase, and bimolecular fluorescence complementation. Also, an immunoblot showed that *Os*FLO6 protein abundance decreased at high temperatures in *oshsp60-3b* anthers compared with the wild type, supporting the role of *Os*HSP60-3B in maintaining starch synthesis by stabilizing *Os*FLO6. Also, *Os*HSP60-3B localized to the plastid stroma in a diffuse pattern, which resembled the known localization of *Os*FLO6 (Zhang et al. 2022). Based on these findings, the authors propose that *Os*HSP60-3B stabilizes *Os*FLO6 to maintain starch synthesis under high-temperature conditions (>34 °C).

**Figure 1.**
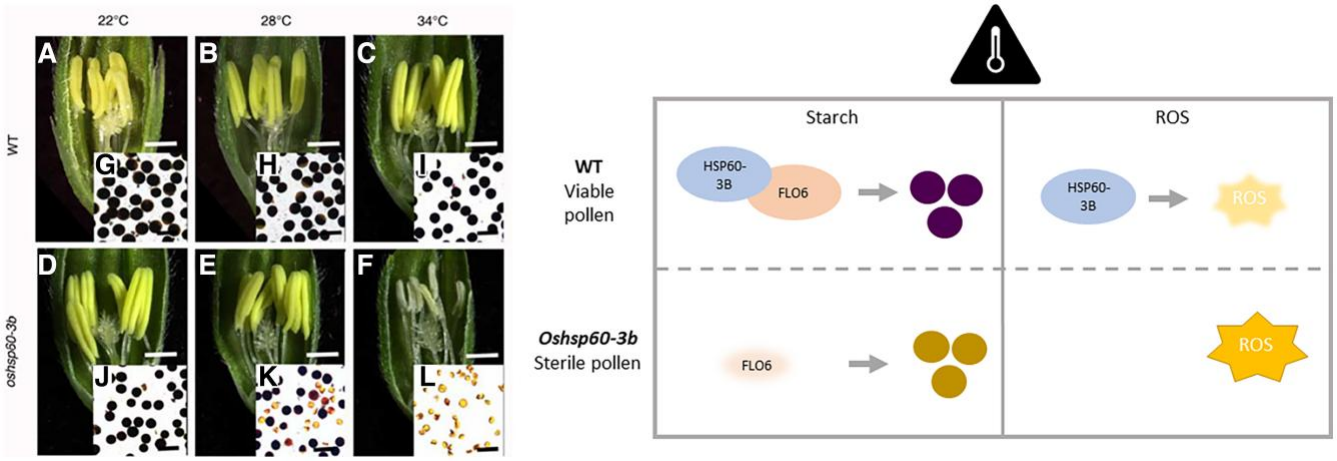
*Os*HSP60-3B ensures pollen viability under heat stress by regulating starch synthesis and ROS levels. Left: Rice anthers and pollen in *oshsp60-3b* mutants resemble wild type (WT) at ambient temperatures, but at high temperatures, anthers become pale and shrivelled and the pollen becomes inviable and loses starch accumulation. This image was taken from Lin et al. (2023). Right: Summary of the 2 functions of *Os*HSP6-3B under heat stress to ensure starch synthesis by stabilizing *Os*FLO6 and to prevent accumulation of ROS through a yet unknown mechanism.

Another interesting possibility raised by this work is that *Os*HSP60-3B may prevent the accumulation of ROS and cell death at high temperatures. The authors performed a comparative RNA-sequencing analysis of *oshsp60-3b* and the wild type at 3 different anther developmental stages at 34 °C and found 298 differentially expressed genes between the mutant and wild type common to all 3 stages. Gene Ontology analysis revealed that oxidation reduction was the most enriched biological process and oxidoreductase activity the most enriched molecular function at all 3 developmental stages. Trypan blue and 3,3′-diaminobenzidine staining confirmed that *oshsp60-3b* had increased H_2_O_2_ accumulation and cell death at high temperatures compared with the wild type.

The findings presented here suggest that *Os*HSP60-3B is a unique HSP involved in the rice male gametophyte heat stress response. Lin et al. (2023) have identified 2 pathways through which this response is regulated: starch synthesis and ROS levels (Fig. 1). Crucially, the authors also found that overexpression of *Os*HSP60 improves rice pollen heat tolerance. Further exploration of the molecular mechanisms could reveal the specificity of *Os*HSP60-3B regulation, such as whether other starch synthesis proteins are also stabilized by *Os*HSP60-3B, how *Os*HSP60-3B prevents excessive ROS accumulation, and whether there are additional *Os*HSP60-3B heat response pathways that have yet to be identified. Further studies should explore how the different subunits of Cpn60s function together to regulate protein folding under various environmental conditions.
